# Medicinal Plant Rhizospheres as Reservoirs of *Aspergillus*-Derived Phytochemicals with Antimicrobial and Insecticidal Potential

**DOI:** 10.3390/life15121886

**Published:** 2025-12-10

**Authors:** Sidra Farooq, Asif Mehmood, Nasir Ali, Amjad Khan, Naeem Khan

**Affiliations:** 1 Sarhad Institute of Allied Health Sciences, Sarhad University of Science and Information Technology, Peshawar 25000, Pakistan; 2 Department of Health & Biological Sciences, Abasyn University, Peshawar 25000, Pakistan; 3 Agronomy Department, University of Florida, Gainesville, FL 32611, USA

**Keywords:** bioactive, fungi, metabolites, GCMS, 18s rRNA

## Abstract

The rhizosphere, a dynamic interface shaped by plant root exudates, fosters microbial communities with significant biochemical potential. This study investigated the interplay between soil properties and fungal bioactivity in the rhizospheres of *Withania coagulans* and *Justicia adhatoda* in Pakistan. Physicochemical analysis revealed silty loam textures with divergent phosphorus [25.7 vs. 71.5 mg/kg] and potassium [108 vs. 78 mg/kg] levels, alongside near-neutral pH, influencing microbial dynamics. Two fungal isolates, *Aspergillus luchuensis* and *A. flavus*, were identified through morphological traits and ITS-region sequencing. Gas chromatography-mass spectrometry [GC-MS] profiling of ethyl acetate extracts uncovered 30 and 25 previously uncharacterized metabolites in *A. luchuensis* and *A. flavus*, respectively, including bioactive compounds such as tetradecanoic acid and nonadecane. Bioassays demonstrated broad-spectrum efficacy against multidrug-resistant clinical isolates, with *A. flavus* exhibiting notable inhibition against *Salmonella typhi* [31.7 mm zone] and *A. luchuensis* against *Shigella* spp. [23 mm]. Both extracts suppressed *Lemna minor* growth by 70%, indicating phytotoxic potential, and displayed species-specific insecticidal activity, inducing 70% mortality by *A. luchuensis* against Blattodea and 50% by *A. flavus* against the same species. These findings underscore the rhizosphere’s role as a reservoir of bioactive fungi, with *Aspergillus* spp. producing metabolites of pharmaceutical and agrochemical relevance. The study highlights the necessity for advanced structural elucidation and ecotoxicological assessments to harness these compounds, advocating integrated approaches combining metabolomics and genomic mining to unlock novel biotechnological applications.

## 1. Introduction

The rhizosphere—the thin layer of soil immediately surrounding plant roots—is enriched by root exudates such as sugars, amino acids, and organic acids, which create nutrient-rich microzones that sustain dense microbial populations and heightened biochemical activity [1]. These “hotspots” exhibit accelerated rates of organic matter decomposition and nutrient turnover compared to bulk soil, driving key processes like nitrogen mineralization and carbon cycling [2].

Soil physicochemical properties—including pH, organic carbon content, and the concentrations of nitrogen [N], phosphorus [P], and potassium [K]—strongly shape rhizosphere microbial community structure and function. Soil pH alone can account for up to 70% of the variation in bacterial diversity across different ecosystems, with neutral-pH soils supporting the greatest richness [3]. Moreover, localized pulses of labile carbon and nutrients at the root–soil interface generate “hot moments” of microbial activity that disproportionately influence overall soil biogeochemistry [2].

Fungi are central to soil ecosystem functioning: their extracellular enzymes decompose complex polymers such as cellulose, lignin, and chitin into simpler compounds available to plants and other microbes [4,5]. Beyond nutrient cycling, many soil fungi produce structurally diverse secondary metabolites—including antibacterial, antifungal, antioxidant, and anticancer agents—that hold promise for pharmaceutical and agricultural applications [6]. In addition, certain fungal metabolites exhibit potent insecticidal activities, offering environmentally friendly alternatives to synthetic pesticides [7].

Within the genus *Aspergillus*, *A. luchuensis* is valued in fermentation industries for its high yields of amylolytic and proteolytic enzymes and prolific citric acid secretion, which lowers mash pH to suppress contaminants [8]. Genome sequencing of strain NBRC 4314 has revealed an extensive suite of glycosidase genes and regulatory networks that underlie its robustness in awamori and shōchū fermentations [8]. In contrast, *A. flavus* is best known for aflatoxin biosynthesis, yet its genome harbors at least 56 cryptic secondary metabolite gene clusters encoding polyketide synthases, non-ribosomal peptide synthetases, and terpene cyclases, many of which can be activated under specific environmental stimuli [9].

The main objectives of this study were to: (1) use GC-MS to profile the extracellular metabolite composition of two *Aspergillus* isolates that were recovered from the rhizospheres of *Withania coagulans* and *Justicia adhatoda*; (2) assess the antimicrobial, phytotoxic, and insecticidal activities of these crude extracts; and (3) investigate relationships between soil physicochemical characteristics and fungal metabolic output. It was possible to identify bioactive candidates of pharmacological or agrochemical interest for further structural and functional validation by connecting metabolite patterns to rhizosphere conditions.

## 2. Materials and Methods

### 2.1. Soil Sampling and Processing

Rhizospheric soil samples were collected from the rhizosphere of *Withania coagulans* and *Justicia adhatoda* cultivated in Bara, District Khyber, Khyber Pakhtunkhwa, Pakistan. Samples were excavated at a depth of 15 cm using sterilized tools, transferred in aseptic polyethylene containers, and transported to the Microbiology Laboratory at Sarhad University of Science and Information Technology, Peshawar, for subsequent analysis.

### 2.2. Physicochemical Profiling of Soil

Temperature, pH, organic carbon (C), nitrogen (N), phosphorus (P), potassium (K), and soil texture were all assessed. The Bouyoucos hydrometer method (Model 152H, Soiltest Inc. Wheaton, IL, USA; accuracy ±0.5%) was used to measure the particle size distribution of sand, silt, and clay. The Soil and Water Analysis Facility, Agricultural Research Institute, Tarnab, Peshawar, Pakistan, quantified organic carbon and nitrogen in accordance with recognized protocols [10]. A UV-Visible spectrophotometer (Model UV-1800, Shimadzu, Japan; accuracy ±0.3 nm) and a flame photometer (Model PFP7, Jenway, Dunmow, Essex, UK; precision ±1%) were used to measure the amounts of potassium and phosphorus, respectively. A portable digital pH meter (Model HI99121, Hanna Instruments Inc., Smithfield, RI, USA; resolution ±0.01 pH) was used to record the initial soil color and pH at the time of collection.

### 2.3. Fungal Isolation and Purification

The plates were incubated under static conditions for 6–8 days at 28 °C. The reason for choosing this temperature is that mesophilic soil fungi typically thrive around 25 to 30 °C, and 28 °C is a typical compromise that allows for the best growth of environmental *Aspergillus* species as well as other rhizosphere fungi. Due to its ability to facilitate the sporulation and growth of a wide variety of soil fungus, Potato Dextrose Agar (PDA) (Thermo Scientific™ Oxoid™. CM0139B, Waltham, MA, USA) was employed. Static aerobic conditions were used for incubation, and the medium was treated with 50 µg/mL of chloramphenicol to promote fungal isolation over bacterial isolation. Pure cultures were produced by isolating the hyphal tips and repeatedly subculturing individual colonies onto new PDA. Prior to DNA extraction, uniform colony morphology and microscopic examination with lactophenol cotton blue were used to verify purity.

### 2.4. Microscopic Characterization

Hyphal and spore structures were analyzed using light microscopy. Slide cultures were prepared to observe reproductive features, including conidiophores and sporangia. Colony morphology [pigmentation, texture] and purity were documented via macroscopic and microscopic examination [10].

### 2.5. Molecular Phylogenetic Analysis

Using the CTAB-based method of [11], genomic DNA was isolated from fresh mycelia using a SolGent Fungus Genomic DNA Extraction Kit (Cat. No. SGD64-S120; SolGent Co., Daejeon, Republic of Korea). By using 1% agarose gel electrophoresis, the purity of the DNA was verified. Since the Internal Transcribed Spacer (ITS) region of nuclear rDNA offers better species-level resolution and functions as the universal fungal barcode in contrast to the more conserved 18S rRNA gene, it was amplified using universal primers ITS1 and ITS4. EF-Taq polymerase (SolGent, Republic of Korea) was used for PCR amplification on a BIO-RAD T100™ thermocycler. The BigDye™ Terminator v3.1 Kit was used to sequence the purified amplicons on an ABI 3730xl DNA Analyzer (Applied Biosystems, Waltham, MA, USA). To identify the obtained sequences, similarity searches were performed using BLAST (Basic Local Alignment Search Tool), NCBI BLAST+ version 2.17.0. 

### 2.6. Metabolite Extraction and GC–MS Profiling

For ten days, fungal isolates were cultivated in Potato Dextrose Broth [PDB] (Thermo Scientific™ Oxoid™. CM0962B) at 28 °C and 150 rpm. Cultures were filtered through Whatman No. 1 filter paper, and the resulting biomass-free filtrates (cell-free supernatants) were collected and processed with Ethyl acetate (Sigma-Aldrich, Saint Louis, MI, USA; CAS 141-78-6) for 24 h in a separatory funnel in 1:1 *v*/*v*. For GC-MS analysis, the organic phase was reconstituted in minimum EtOAc after being concentrated by rotational evaporation at 40 °C and 150 mbar. Chromatographic separation was carried out using an Agilent 7890B GC system (Agilent Technologies, Santa Clara, CA, USA) that was interfaced with a 5977B mass selective detector (Agilent Technologies, USA; mass accuracy ±0.1 m/z) and fitted with a DB-5MS capillary column (30 m × 0.25 mm, 0.25 μm film thickness; J&W Scientific, Santa Clara, CA, USA). The carrier gas was helium (99.999%, purity certified, Linde Gas, Pullach, Germany) at a steady 1 mL/min flow rate. Ionization was carried out at 70 eV. Metabolites were annotated using the NIST 2020 library [12]. Following solvent partitioning, the ethyl acetate fractions were combined and dried by rotary evaporation and vacuum desiccation to a consistent weight at a lower pressure. Prior to bioassay, the dry crude extracts were weighed and kept at −20 °C. Extracts were reconstituted in less than 1% DMSO to provide stock solutions for bioactivity tests.

Ethyl acetate was solely used to extract the culture filtrate in this investigation; the mycelial biomass was not included. Extracellular metabolites were the main focus of study, since they are released into the medium and are more likely to be diffusible bioactive substances that affect rhizosphere interactions. Mycelial extraction usually targets intracellular metabolites that require acetone or methanol extraction and subsequent cleanup, while extracellular extracts frequently contain higher concentrations of volatile and low-molecular-weight compounds amenable to GC–MS analysis, according to previous reports.

### 2.7. Detection of Multidrug-Resistant [MDR] Bacterial Species

Clinical specimens from diabetic foot ulcers were obtained from City General Hospital, Peshawar, and streaked on nutrient agar. Isolates were identified through Gram staining, colony morphology, and biochemical assays [catalase, oxidase, indole, citrate utilization, TSI]. Antibiotic susceptibility was tested against 10 broad-spectrum antibiotics [Table 1] [13] on Mueller-Hinton agar via disk diffusion [CLSI, 2020]. MDR strains resistant to ≥3 antibiotic classes were selected for bioactivity assays [14].

### 2.8. Antimicrobial Bioassays

Crude extracts [3 mg/mL DMSO] were tested in triplicate (n = 3) against multidrug-resistant bacterial and fungal isolates using the agar-well diffusion method. Wells [6 mm] were loaded with 100 µL extract, with ciprofloxacin [5 µg/mL] and fluconazole [25 µg/mL] as positive controls. The mean diameter (mm ± SE) of inhibition zones was recorded after 24–48 h incubation at 37 °C. Minimum inhibitory concentration (MIC) values were determined via broth microdilution (range = 0.01–0.64 µg/mL), and IC_50_ values were calculated using % mortality [15]. The usual starting points for assessing the bioactivity of crude extracts in natural product research were determined to be 3 mg/mL for antibacterial and 4 mg/mL for antifungal tests. These concentrations are frequently employed to identify activity in unfractionated crude material.

### 2.9. Phytotoxicity Assessment

*Lemna minor* fronds were cultured in E-media supplemented with 100 µg/mL extract [1 mg/mL stock in DMSO]. Growth inhibition was quantified after 7 days at 30 °C relative to paraquat-treated [positive control (1 mg/mL)] and untreated groups and the assays were performed in triplicate [16]. Frond viability was calculated as:

$$Growth\;Inhibition[\%]=\left(\frac{No.of\;fronds\;affected\;in\;sample}{Total\;number\;of\;fronds\;in\;control}\right)\times 100$$

### 2.10. Insecticidal Bioactivity

Termites [*Isoptera*] and cockroaches [*Blattodea*] were exposed to filter paper impregnated with 100 µg culture extract /mL DMSO [17]. All assays were performed in triplicate. Permethrin (0.1 mg/mL) was used as positive control for insecticidal tests. Extract concentrations ranged from 10 to 100 µg/mL to calculate IC_50_ values. Mortality percentages were expressed as mean ± SE.

$$\%Mortality=\left(\frac{d}{n}\right)\times 100$$

### 2.11. Statistical Evaluation

Data were analyzed using SPSS 16.0 [SPSS Inc., Chicago, IL, USA]. Mean ± standard error [SE] was calculated for triplicate experiments.

## 3. Results

### 3.1. Physicochemical Properties of Rhizosphere Soils

The rhizosphere soils of *Withania coagulans* and *Justicia adhatoda* exhibited a silty loam texture with distinct quantitative differences. The sand content was 54% in *W. coagulans* soil compared to 64% in *J. adhatoda* soil, while clay and silt contents were comparable [12% vs. 10% for clay and 26% for silt in both soils]. Organic matter [1.03% vs. 1.06%] and nitrogen levels [0.052 mg/kg vs. 0.053 mg/kg] were nearly identical between the two soils. However, phosphorus concentrations diverged significantly, with *W. coagulans* soil containing 25.7 mg/kg versus 71.5 mg/kg in *J. adhatoda* soil, and potassium levels were higher in *W. coagulans* soil [108 mg/kg] compared to *J. adhatoda* soil [78 mg/kg]. Both soils exhibited a slightly acidic to neutral pH, consistent with fertile brown soils [Table 2].

### 3.2. Isolation and Morphological Characterization of Rhizospheric Fungi

**Figure 1:**
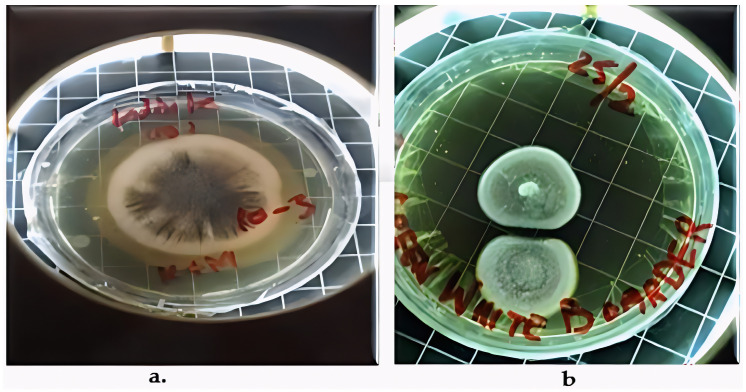
Colony Morphology of Rhizospheric Fungi on PDA potato dextrose agar. (**a**) *Aspergillus luchuensis* (MTCC strain) from *Justicia adhatoda* exhibits a distinctive black-and-white mycelial pattern with sporangia; (**b**) *Aspergillus flavus* (WTCC strain) from *Withania coagulans* displays olive-green conidia with white margins.

**Figure 2:**
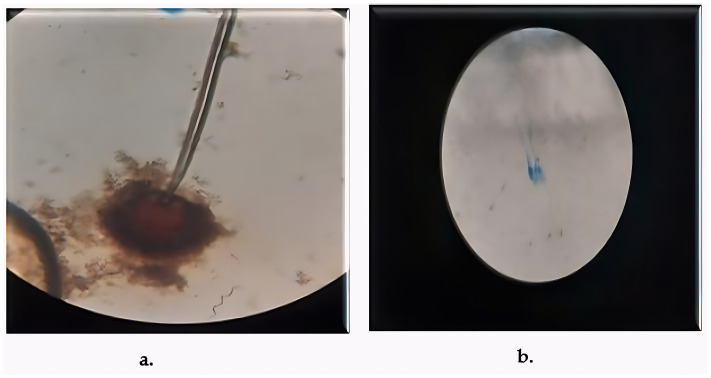
Microscopic Structures of Rhizospheric Fungi Light micrographs of fungal isolates stained with lactophenol cotton blue. (**a**) *A. luchuensis* showing sporangia with clustered spores; (**b**) *A. flavus* exhibiting branched conidiophores with powdery conidial heads.

Two fungal strains, *Aspergillus luchuensis* and *Aspergillus flavus,* were isolated from the rhizospheres. On PDA, *A. luchuensis* developed colonies with a distinctive black-and-white mycelial pattern and formed sporangia containing coenocytic hyphae [Figure 1]. In contrast, *A. flavus* produced colonies characterized by olive-green conidia with white margins and displayed branched conidiophores bearing powdery conidial heads [Figure 1 and Figure 2].

### 3.3. Molecular Identification via 18S rRNA Sequencing

**Figure 3:**
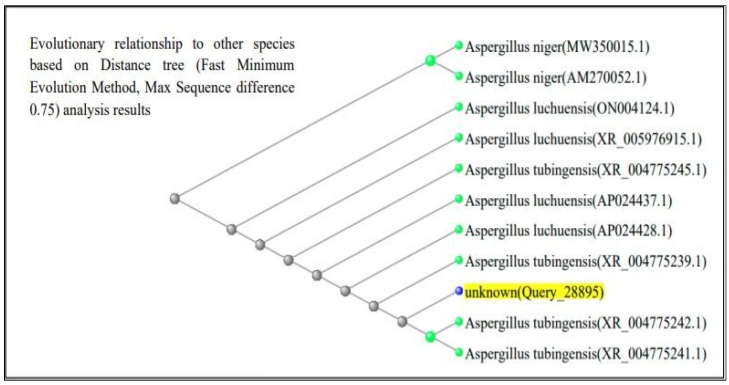
Sequence analysis of *Aspergillus luchuensis* (MTCC strain). BLAST alignment confirmed 99–100% sequence similarity with reference strains of *A. luchuensis* (NCBI accession: PQ571950), supporting its molecular identification.

**Figure 4:**
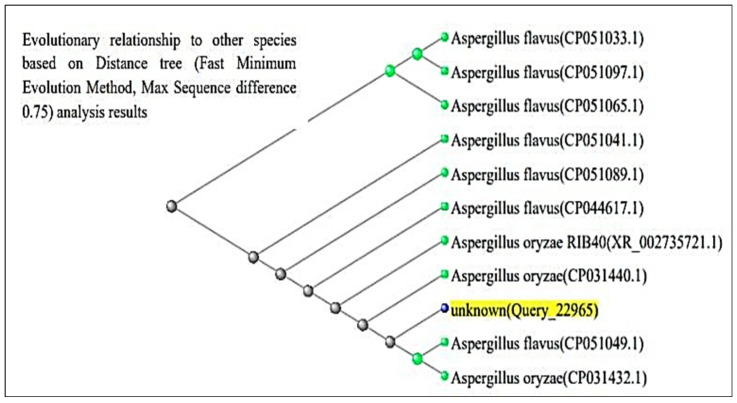
The sequence of *A. flavus* (WTCC strain) is presented. Homology analysis demonstrated 100% similarity with the reference strain (NCBI accession: PQ571952), verifying its genetic identity.

The ITS region of nuclear rDNA from both fungal isolates was amplified using universal primers ITS1 and ITS4, and the resulting sequences were analyzed using the BLAST tool in the NCBI GenBank database. According to Figure 3 and Figure 4, BLAST analysis confirmed that both isolates shared 99–100% sequence similarity with their respective *Aspergillus* reference strains. The *A. flavus* isolate showed 100% sequence identity with *A. flavus* reference sequences (GenBank accession no. PQ571952), while the *A. luchuensis* isolate showed 99–100% sequence similarity with *A. luchuensis* reference sequences (GenBank accession no. PQ571950). These accession numbers correspond to the sequences submitted to GenBank following verification of full homology, ensuring taxonomic accuracy.

### 3.4. GC–MS Profiling of Fungal Culture Filtrates

**Figure 5:**
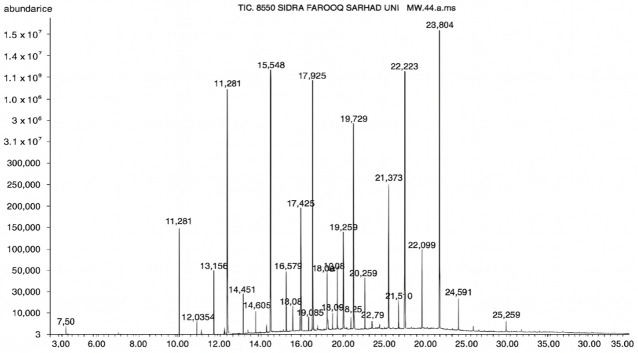
GC–MS chromatogram of the ethyl acetate extract from *A. luchuensis* is shown, highlighting peaks corresponding to 30 hitherto unreported compounds reported for the first time from this source. Retention times and relative intensities are displayed, reflecting a complex metabolic profile.

**Figure 6:**
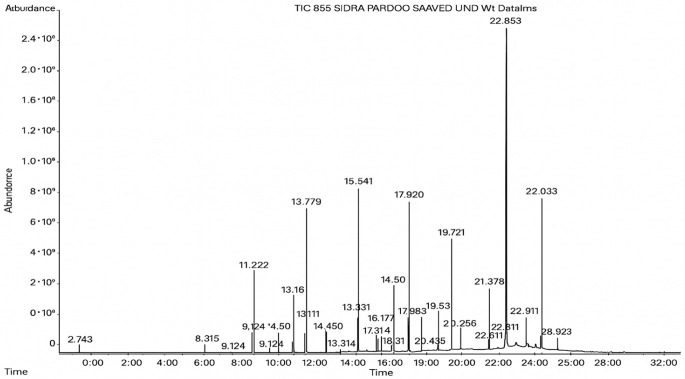
The GC–MS profile of the *A. flavus* ethyl acetate extract revealed 25 hitherto unreported compounds reported for the first time from this source, visualized through distinct retention peaks. The data support the metabolic versatility of the isolate.

GC–MS analysis identified 30 and 25 compounds in *A. luchuensis* and *A. flavus*, respectively. Only compounds with a library match quality above 90% were included, and their relative proportions were calculated from normalized peak areas in the total ion chromatogram. Among the novel compounds detected in both extracts were p-Xylene, tetradecane, and bis[2-ethylhexyl] phthalate (Figure 5 and Figure 6). Additionally, known bioactive compounds such as tetradecanoic acid, which has documented antifungal and antioxidant properties, and nonadecane, noted for its antimicrobial activity, were also identified [Table 3, Table 4, Table 5 and Table 6].

**Table 3:** Hitherto unreported compounds identified for the first time from the crude ethyl acetate extract of *Aspergillus luchuensis*.

S. No	Peak No.	Retention Time (Mins)	Peak Areas	Reference Peaks	Quality	Structures	Functions	References
1.	1	2.750	10,288,308	5721	95	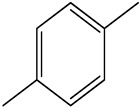 p-Xylene	Solvent in printing and painting industries	[18]
2.	2	11.281	78,790,005	74,004	98	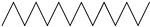 Tetradecane	Antibacterial and antifungal agent	[19]
3.	3	12.098	16,323,498	90,197	96	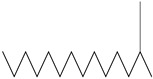 Tetradecane, 2-methyl-	Function unknown till now	
4.	4	12.542	17,945,463	90,192	97	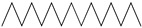 Pentadecane	Antifungal agent	[20]
5.	5	13.158	56,062,405	101,093	98	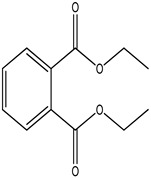 Diethyl Phthalate	Antibacterial agent	[21]
6.	6	13.612	28,625,634	104,590	99	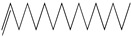 Cetene	Function unknown till now	
7.	7	13.754	240,438,495	107,219	99	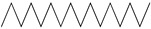 Hexadecane	Function unknown till now	
8.	8	14.461	36,352,485	123,967	98	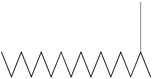 Hexadecane, 2-methyl	Function unknown till now	
9.	9	14.856	26,610,402	123,958	98	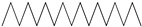 Heptadecane	Improves oxidative stress-related diseases	[22]
10.	10	15.318	17,507,832	109,284	98	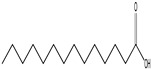 Tetradecanoic acid	Antifungal, antioxidant, anticancer, nematocidal and also functions as lubricant	[23]
12.	12	15.948	293,848,711	141,057	97	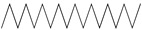 Octadecane	Function unknown till now	
13.	17	17.639	7,140,310	141,058	96			
14.	32	20.556	11,375,318	141,058	96			
15.	14	16.579	52,483,248	158,611	98	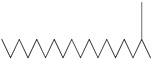 Octadecane, 2-methyl-	Antimicrobial and antitumor potential	[24]
16.	15	15.501	24,273,045	158,597	98	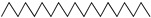 Nonadecane	Activity against plant fungal pathogens	[25]
17.	16	17.428	233,542,838	143,511	99	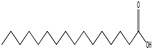 n-Hexadecanoic acid	Antioxidant, nematicide and pesticide	[26]
18.	18	17.690	13,409,546	179,135	98	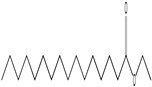 Hexadecanoic acid, ethyl ester	Antioxidant, hemolytic, hypo-cholesterolemic, nematicide and anti-androgenic	[27]
19.	19	17.816	15,840,959	138,521	99	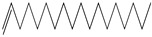 1-Octadecene	Medicines and cosmetics	[28]
20.	20	17.925	258,633,377	176,384	98	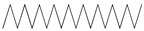 Eicosane	Antimicrobial activities	[29]
21.	31	20.259	42,778,282	176,387	93			
22.	45	26.989	17,988,115	176,384	98			
23.	22	18.503	53,705,129	194,594	99	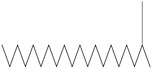 Eicosane, 2-methyl-	Bioactive compound	[30]
24.	23	18.824	17,867,516	194,588	98	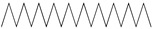 Heneicosane	Pheromonic and fumigating properties	[29]
25.	24	18.935	98,703,795	173,581	99	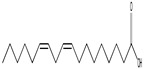 9,12-Octadecadienoic acid (Z,Z)-	Antimicrobial properties	[26]
26.	28	19.520	8,277,967	214,945	98	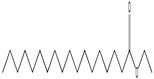 Octadecanoic acid, ethyl ester	Antimicrobial agent	[31]
27.	34	21.304	9,835,708	209,740	99	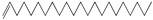 1-Docosene	Antibacterial compound	[32]
28.	35	21.379	117,296,495	246,483	99	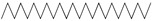 Tetracosane	Antagonistic potential against toxigenic and phytopathogenic fungi	[33]
29.	43	24.591	33,753,194	246,483	99			
30.	36	21.876	35,975,300	262,016	99	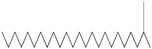 2-Methyltetracosane	Function unknown till now	
31.	37	22.160	7,134,674	262,011	98	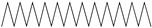 Pentacosane	Function unknown till now	
32.	38	22.229	282,707,118	295,611	91	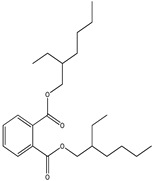 Bis(2-ethylhexyl) phthalate	Antibacterial and larvicidal activity	[34]
33.	40	22.908	69,037,303	275,538	98	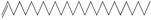 Hexacosene	Function unknown till now	
34.	41	23.390	20,703,676	246,483	98	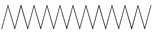 Tetracosane	Antagonistic potential against toxigenic and phytopathogenic fungi	[33]
35.	42	23.904	445,455,491	295,785	94	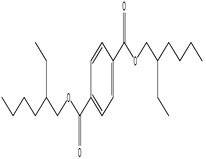 1,4-Benzenedicarboxylic acid, bis(2-ethylhexyl) ester	Function unknown till now	
36.	44	25.259	12,351,665	307,034	99	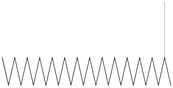 Octacosane, 2-methyl-	Antimicrobial agent	[31]

**Table 4:** Reported Compounds Extracted from Crude Ethyl Acetate Extract of *A. luchuensis.*

S. No	Peak No.	Retention Time (Mins)	Peak Areas	Reference Peaks	Quality	Structures	Functions	References
1.	25	19.009	77,699,002	176,208	99	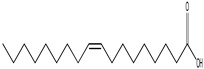 Oleic Acid	Function unknown till now	
2.	26	19.182	19,343,950	209,617	99	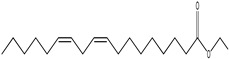 Linoleic acid ethyl ester	Function unknown till now	
3.	27	19.257	133,934,985	179,093	99	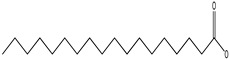 Octadecanoic acid	Function unknown till now	
4.	29	19.635	15,910,485	155,961	98	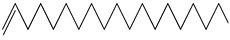 1-Nonadecene	Antifungal and anticancer agent	[26]
5.	30	19.726	181,536,736	212,282	99	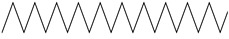 Docosane	Function unknown till now	
6.	39	22.846	7,637,629	273,800	99	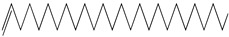 1-Hexacosene	Function unknown till now	

**Table 5:** Hitherto unreported compounds identified for the first time from the crude ethyl acetate extract of *Aspergillus flavus.*

S. No	Peak No.	Retention Time (Mins)	Peak Areas	Reference Peaks	Quality	Structures	Functions	References
1.	1	2.749	9,481,682	5720	97	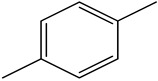 p-Xylene	Solvent in printing and painting industries	[18]
2.	2	8.895	48,805,295	18,696	94	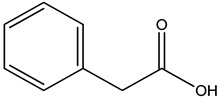 Benzene acetic acid	Broad-spectrum antifungal agent	[35]
3.	3	9.912	6,729,875	59,028	95	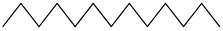 Tridecane	Antioxidant activity	[36]
4.	5	11.131	34,797,276	71,738	99	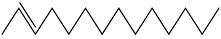 2-Tetradecene, (E)-	Anticancer, antimicrobial and antioxidant	[37]
5.	6	11.282	104,087,172	74,004	98	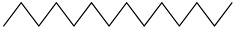 Tetradecane	Antibacterial and antifungal agent	[19,38]
6.	7	11.869	10,474,783	99,032	98	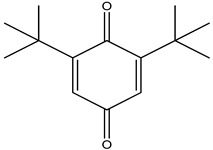 2,5-Cyclohexadiene-1,4-dione, 2,6-bis (1,1- dimethylethyl)-	Antibacterial and antifungal agent	[19,38]
7.	8	12.099	18,795,488	74,012	90	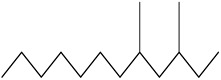 3,5-Dimethyldodecane	Function unknown till now	
8.	9	12.425	32,616,847	82,737	97	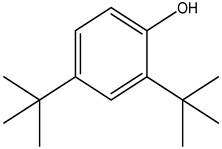 2,4-Di-tert-butylphenol	Exhibits strong toxicity against significant ratio of tested organisms as well as the producing species	[39]
9.	10	12.541	17,844,280	90,192	97	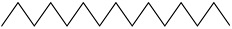 Pentadecane	Antifungal agent	[20]
10.	11	13.161	80,026,380	101,097	98	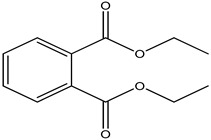 Diethyl Phthalate	Active against Gram-positive and Gram-negative bacterial species	[21]
11.	12	13.614	56,298,031	104,590	99	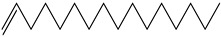 Cetene	Function unknown till now	
12.	16	15.317	15,633,869	109,281	96	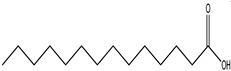 Tetradecanoic acid	Antifungal, antioxidant, anticancer, nematocidal, hypercholesterolemic and can be used as lubricant	[23]
13.	19	16.194	13,005,006	241,696	90	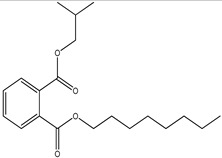 Phthalic acid, isobutyl octyl ester	Anti-microbial activity	[40]
14.	22	16.709	19,568,824	168,692	99	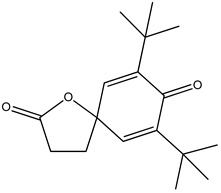 7,9-Di-tert-butyl-1-oxaspiro (4,5) deca-6,9-diene-2,8- dione	Prevention and treatment of periodontitis	[41]
15.	23	16.933	22,968,819	158,600	98	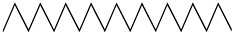 Nonadecane	Active against plant fungal pathogens	[25]
16.	24	17.106	22,346,257	170,787	95	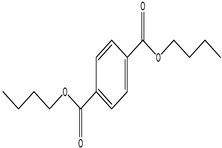 1,4-Dibutyl benzene-1,4-dicarboxylate	Function unknown till now	
17.	30	18.824	13,405,806	194,587	98	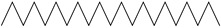 Heneicosane	Antifungal compound	[42]
18.	33	19.635	46,586,432	209,741	99	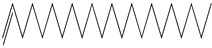 Docosene	Function unknown till now	
19.	34	19.721	150,288,747	212,282	98		Function unknown till now	
20.	37	21.304	25,049,939	244,233	99	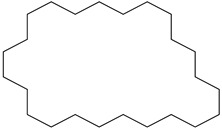 Cyclotetracosane	Antibacterial as well as α-amylase inhibitory activity	[43]
21.	38	21.376	95,053,436	246,483	99	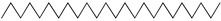 Tetracosane	Antagonistic potential against toxigenic and phytopathogenic fungi	[33]
22.	39	21.872	49,711,235	262,016	99	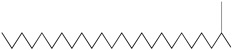 2-Methyltetracosane	Function unknown till now	
23.	40	22.293	1,231,442,386	295,608	91	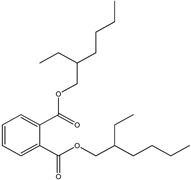 Bis(2-ethylhexyl) phthalate	Antibacterial and larvicidal activity	[34]
24.	41	22.850	16,685,206	259,983	99	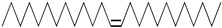 Z-12-Pentacosene	Function unknown till now	
25.	44	25.259	12,351,665	295,779	95	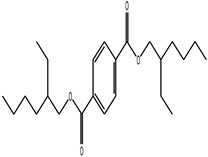 1,4-Benzenedicarboxylic acid, bis(2-ethylhexyl) ester	Function unknown till now	
26.	45	23.979	13,522,101	245,085	97	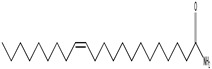 13-Docosenamide, (Z)-	Function unknown till now	

**Table 6:** Reported Compounds Extracted from Crude Ethyl Acetate Extract of *A. flavus.*

S. No	Peak No.	Retention Time (Mins)	Peak Areas	Reference Peaks	Quality	Structures	Functions	References
1.	4	11.068	22,425,688	22,402	94	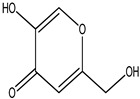 4H-Pyran-4-one, 5-hydroxy-2-(hydroxymethyl)	Natural kojic acid	[44]
2.	13	13.749	202,662,852	107,219	99	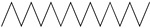 Hexadecane	Function unknown till now	
3.	14	14.460	30,280,445	123,967	99	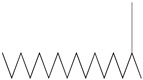 Hexadecane, 2-methyl	Function unknown till now	
4.	15	14.854	22,603,766	123,960	98	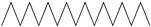 Heptadecane	Improves oxidative stress-related diseases	[22]
5.	17	15.821	54,267,257	138,490	99	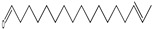 E-15-Heptadecenal	Antifungal, anti-cancerous, anti-inflammatory and antioxidant properties	[45]
6.	18	15.941	239,852,366	141,058	98	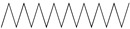 Octadecane	Function unknown till now	
8.	36	20.555	6,645,236	141,056	95			
9.	46	24.593	22,146,253	141,056	96			
10.	20	16.521	21,799,447	87,012	97	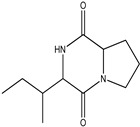 Pyrrolo[1,2-a]pyrazine-1,4-dione, hexahydro-3-(2- methylpropyl)-	Function unknown till now	
11.	21	16.577	50,622,981	158,611	97	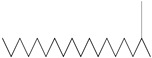 Octadecane, 2-methyl	Function unknown till now	
12.	25	17.420	208,755,615	143,511	99	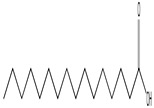 n-Hexadecanoic acid	Antioxidant, nematicide and pesticide	[26]
14.	28	17.920	209,202,265	176,384	99	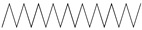 Eicosane	Pheromonic, antimicrobial activities and fumigating properties	[29]
15.	29	18.505	76,888,883	194,594	97	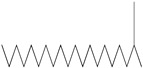 Eicosane, 2-methyl	Bioactive compound	[30]
16.	31	18.970	15,114,772	176,208	99	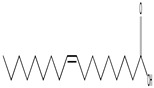 Oleic Acid	Function unknown till now	
17.	32	19.236	88,525,166	179,093	99	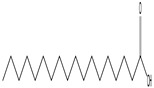 Octadecanoic acid	Function unknown till now	
18.	35	20.256	37,411,957	212,282	97	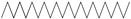 Docosane	Function unknown till now	
19.	42	22.911	49,585,222	275,538	97	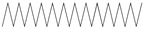 Hexacosane	Function unknown till now	
20.	43	23.392	16,166,875	246,483	97	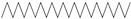 Tetracosane	Antagonistic potential against toxigenic and phytopathogenic fungi	[33]

### 3.5. Antibiotic Resistance Profiling of Diabetic Foot Ulcer Bacteria

**Figure 7:**
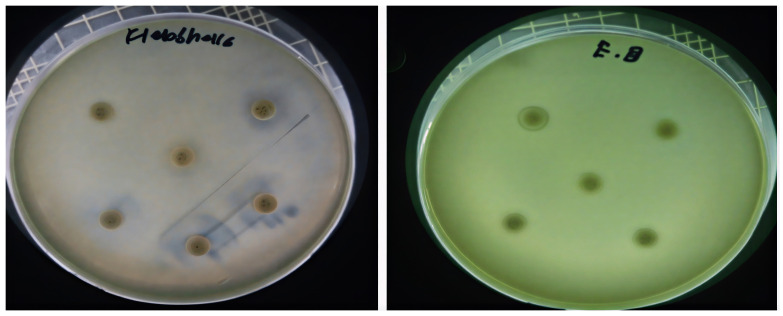
Antibiotic resistance patterns of bacterial isolates recovered from diabetic foot ulcers are depicted. Clear area represents the diameter of inhibition zones for each antibiotic, emphasizing extensive resistance observed among multiple strains.

**Figure 8:**
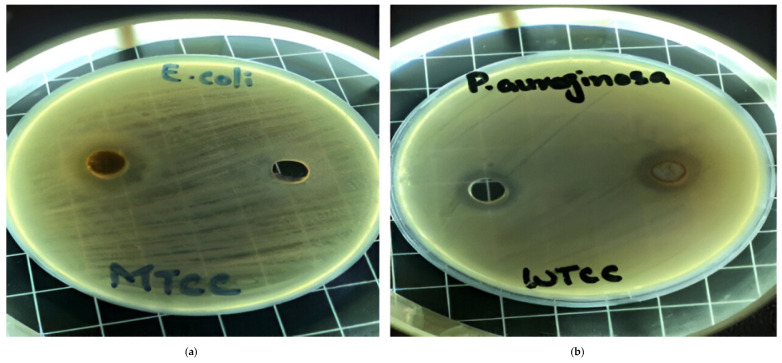
Antibacterial activity of fungal culture filtrates against MDR bacteria. Panel (**a**) illustrates zones of inhibition induced by *A. luchuensis* extract, while panel (**b**) shows enhanced activity by *A. flavus* extract, particularly against *S. typhi*, *S. epidermidis*, and *K. pneumoniae*.

Eight multidrug-resistant [MDR] bacterial species were isolated from diabetic foot ulcer samples and identified through gram staining and biochemical tests, including *Escherichia coli*, *Pseudomonas aeruginosa*, *Klebsiella pneumoniae*, *Shigella* spp., *Salmonella typhi*, *Enterobacter aerogenes*, *Staphylococcus aureus*, and *Staphylococcus epidermidis* [Table 7, Table 8 and Table 9]. Antibiotic susceptibility assays conducted according to CLSI-2020 guidelines revealed resistance to three or more drug classes, with *P. aeruginosa* demonstrating resistance to 9 out of the 10 antibiotics tested [Figure 7 and Figure 8].

### 3.6. Antibacterial Activity of Fungal Extracts

**Figure 9:**
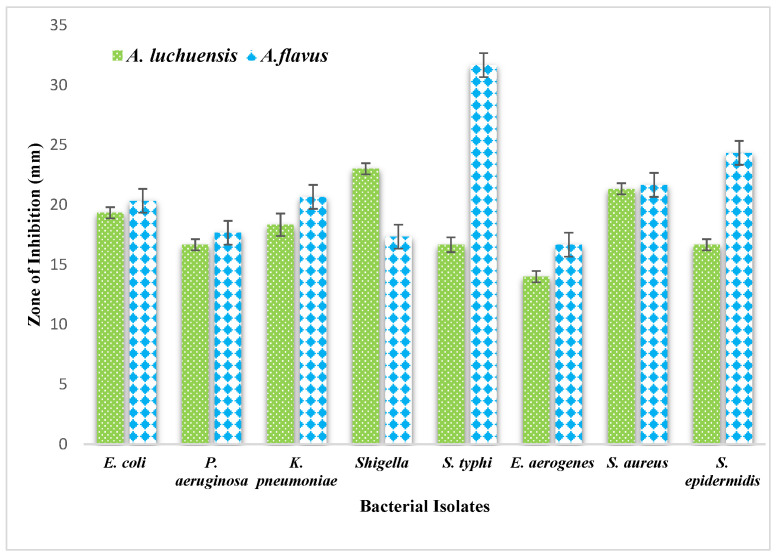
Comparative graphical representation of antibacterial efficacy of *A. luchuensis* and *A. flavus* extracts. Inhibition zones against multiple MDR bacterial strains are shown, with *A. flavus* exhibiting a broader spectrum of activity.

The antibacterial potential of the fungal culture filtrates was evaluated using a 3 mg/mL solution in diluted DMSO. The *A. luchuensis* filtrate exhibited inhibition zones of 23 mm against *Shigella*, 21.33 mm against *S. aureus*, and 19.33 mm against *Enterobacter aerogenes*. In contrast, the *A. flavus* filtrate demonstrated broader antibacterial efficacy, with inhibition zones of 31.66 mm against *Salmonella typhi*, 24.33 mm against *S. epidermidis*, 21.66 mm against *S. aureus*, 20.66 mm against *K. pneumoniae*, and 20.33 mm against *E. coli* [Figure 9].

### 3.7. Antifungal and Phytotoxic Effects

**Figure 10:**
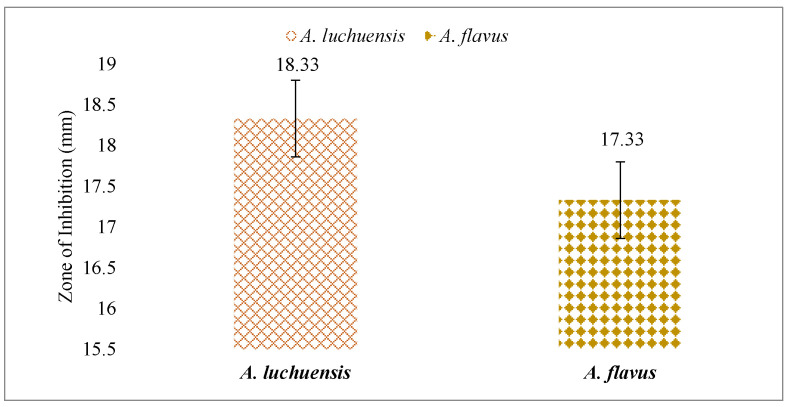
A comparative assessment of antifungal activity between *A. luchuensis* and *A. flavus* is presented. Both extracts showed inhibition against *Candida albicans*, with marginally higher efficacy observed for *A. luchuensis*.

**Figure 11:**
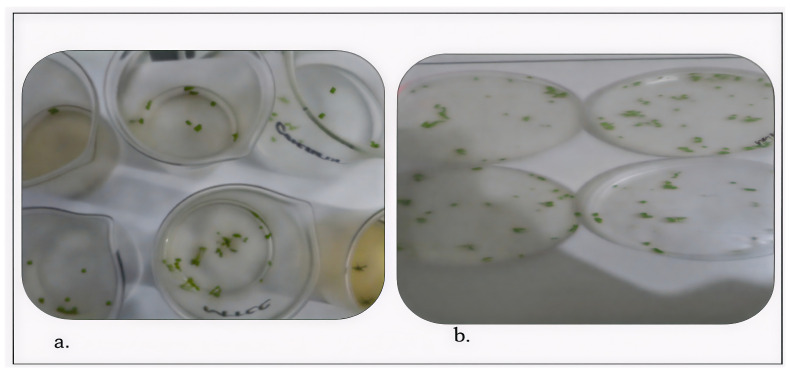
Phytotoxic effects of fungal crude extracts on *Lemna minor* are shown. Panel (**a**) depicts results from *A. luchuensis*, and panel (**b**) from *A. flavus*. Both treatments caused 70% growth inhibition after 7 days of exposure at 100 µg/mL.

Both fungal extracts were tested against *Candida albicans*. The *A. luchuensis* filtrate produced an inhibition zone of 18.33 mm, slightly exceeding the 17.33 mm zone produced by the *A. flavus* filtrate [Figure 10 and Figure 11]. Additionally, when applied at 100 μg/mL, both extracts suppressed the growth of *Lemna minor* by 70%, indicating comparable phytotoxicity (Table 10).

**Table 10:** Gram Staining and Biochemical Characteristics of *Candida albicans* Isolated from Diabetic Foot Ulcers.

Test	Method/Medium Used	Observation/Result
Gram staining	Smear stained with crystal violet–iodine–safranin sequence	Purple oval budding yeast cells
Cell morphology (microscopy)	Wet mount (40×)	Oval to spherical budding cells, pseudohyphae observed
Sugar fermentation	Glucose, maltose, sucrose, lactose broths with Durham tubes	Glucose and maltose fermented with gas; sucrose/lactose not fermented
Urease test	Christensen’s urea agar	No color change (-ive)
Catalase test	3% hydrogen peroxide	Immediate effervescence (+ive)

### 3.8. Insecticidal Potential

**Figure 12:**
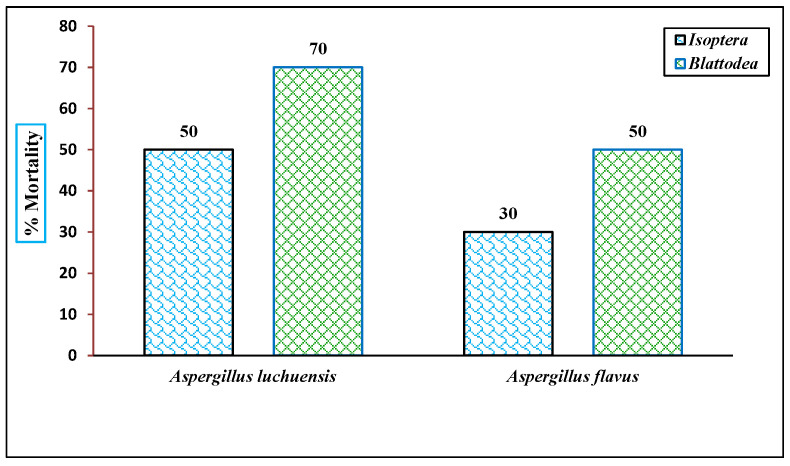
The insecticidal activity of fungal extracts is displayed against *Isoptera* and *Blattodea*. Mortality rates were recorded at 100 µg/mL after 24 h, with *A. luchuensis* showing higher efficacy across both insect taxa.

**Figure 13:**
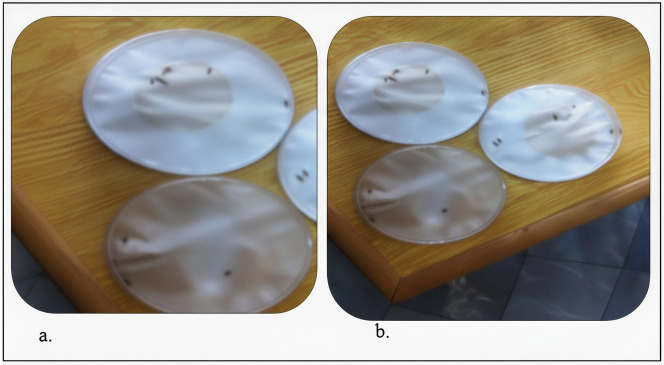
Insecticidal activity of Culture filtrate of (**a**) *A. luchuensis* and (**b**) *A. flavus*

The insecticidal activities of the fungal culture filtrates were evaluated at a concentration of 100 μg/mL against two insect orders. The *A. luchuensis* filtrate induced a mortality rate of 40% in *Isoptera* and 70% in *Blattodea*, while the *A. flavus* filtrate caused 30% mortality in *Isoptera* and 50% in *Blattodea* within 24 h [ Figure 12 and Figure 13]. These results concluded that the insecticidal properties are species-specific, with *A. luchuensis* exhibiting a more pronounced effect.

## 4. Discussion

The differential P and K levels and minor textural variations between the two rhizospheres may have an impact on secondary metabolism and the composition of fungal communities, even though causality cannot be conclusively proved from the current dataset. The different chemical profiles seen for the two isolates may partially reflect local soil chemistry since factors like nutrient status and root exudation patterns might alter the expression of fungal biosynthetic gene clusters. To firmly connect soil properties to biosynthetic output, future research integrating metagenomics, transcriptomics, and targeted metabolomics will be required.

The bioactivities and extracellular metabolite profiles of two rhizospheric *Aspergillus* isolates linked to *Withania coagulans* and *Justicia adhatoda* were evaluated in this investigation. Numerous volatile and non-polar metabolites with documented antibacterial or insecticidal properties were included in the preliminary inventory of metabolites produced by the GC–MS study. The biological effects of crude ethyl acetate extracts included phytotoxicity against *Lemna minor* and detectable inhibition against clinical isolates that were resistant to many drugs. Local soil chemistry, particularly variations in phosphate and potassium levels, may be the cause of the observed variations in metabolite composition between the two isolates. These variations have been shown to affect secondary metabolism in soil fungi. Integration of transcriptomics and metabolomics is still needed to test the mechanistic relationships between soil characteristics and metabolite expression.

Axenic cultures of *A. luchuensis* and *A. flavus* were corroborated by characteristic colony pigmentation and microscopy, and then validated by ITS region sequencing. The genome of *A. luchuensis* NBRC 4314 [~34.7 Mb] encodes extensive glycosidase repertoires and regulatory networks underpinning its robust enzyme secretion [18]. Conversely, *A. flavus* harbors at least 55 cryptic secondary metabolite gene clusters—including polyketide synthases, nonribosomal peptide synthetases, and terpene cyclases—many of which can be activated by environmental cues [19].

GC–MS analyses revealed 30 novel compounds in *A. luchuensis* and 25 in *A. flavus*, such as p-xylene, tetradecane, and bis[2-ethylhexyl] phthalate. Fungal extracellular enzymes decompose complex polymers [cellulose, lignin, chitin] into simpler precursors for secondary metabolism [20,21]. The identification of known bioactive—tetradecanoic acid [antifungal, antioxidant] and nonadecane [antimicrobial]—reinforces the biochemical versatility of rhizosphere fungi.

Crude extracts exhibited broad-spectrum antibacterial effects against multidrug-resistant clinical isolates, with inhibition zones up to 31.7 mm. Fungal secondary metabolites disrupt bacterial cell walls, inhibit protein synthesis, or induce oxidative stress [22]. The pronounced activity of *A. flavus* extracts against *Salmonella typhi* and *Klebsiella pneumoniae* underscores their potential as novel antimicrobials. At 100 µg/mL, both extracts suppressed *L. minor* growth by ~70%, indicating the presence of phytotoxic compounds that may interfere with photosynthetic pigments or hormone signaling. Fungal terpenoids and alkaloids are known to induce oxidative damage and growth inhibition in aquatic plants, suggesting potential for bioherbicide development [23,24,25,26]. *Aspergillus luchuensis* extracts caused 70% mortality in *Blattodea* and 40% in *Isoptera* at 100 µg/mL, outperforming *A. flavus* in certain assays. Fungal metabolites such as indole diterpenoids and tetramic acids target insect nervous systems or digestive enzymes [27,28,29,30], and species-specific efficacy suggests distinct metabolite spectra.

Numerous metabolites that were inferred by GC-MS have been reported to have antibacterial, antioxidant, or insecticidal qualities in the past, which recommended that they may have contributed to the bioactivities that were observed. Tetradecanoic acid and n-hexadecanoic acid, for example, have well-established antibacterial, antifungal, and nematocidal qualities [31,32,33,34]. Both eicosane and nonadecane have showed inhibitory effects on insect pests and phytopathogenic fungi [35,36,37,38]. Similarly, 1,4-benzenedicarboxylic acid bis(2-ethylhexyl) ester and bis(2-ethylhexyl) phthalate showed larvicidal and antibacterial properties [39,40,41,42]. Even though correlation alone cannot prove causation, these activities backed by the literature advised that these metabolites probably have a role in the antibacterial and insecticidal effects seen in this investigation [43,44].

Notably, GC–MS spectrum library matching (NIST 2020) was used for compound identification in this investigation. This method offers a tentative chemical annotation but no conclusive structural confirmation [45]. The identities of the identified metabolites should therefore be regarded as tentative until confirmed by reliable standards or spectroscopic characterization (e.g., ^1^H/^13^C-NMR, HR-MS). This restriction is recognized in order to avoid interpreting the GC-MS data too broadly.

Alkaloids, polyketides, and peptides such gliotoxin, kojic acid, and aspergillic acid are examples of semi-polar or polar secondary metabolites that are typically represented under these analytical circumstances, whereas GC-MS preferentially detects volatile and non-polar chemicals. Such compounds are known to be synthesized by *Aspergillus* species, and they may play a significant role in the observed insecticidal and antibacterial properties.

Activation of silent gene clusters via epigenetic modulators or co-culture can unlock further metabolite diversity [46]. Integrating shotgun metabolomics, genome mining, and bioassay-guided fractionation will accelerate discovery of novel antibiotics, herbicides, and insecticides from rhizosphere fungi.

## 5. Conclusions

This study established a clear link between rhizosphere soil properties and the biosynthetic potential of *Aspergillus luchuensis* and *A. flavus* isolated from the rhizospheres of *Withania coagulans* and *Justicia adhatoda*. Variations in soil phosphorus, potassium, and pH influenced fungal metabolite production, leading to the identification of 55 previously unreported compounds from these sources for the first time through GC–MS analysis. Several metabolites, including tetradecanoic acid and nonadecane, exhibited strong antibacterial, phytotoxic, and insecticidal activities, with inhibition zones up to 31.7 mm against *Salmonella typhi* and 70% mortality against *Blattodea*. These results position rhizospheric *Aspergillus* species as promising biotechnological resources for the development of eco-friendly antimicrobial and agrochemical agents. Future studies integrating metabolomics, genomics, and bioassay-guided fractionation are essential to structurally characterize these compounds and validate their efficacy and safety for sustainable pharmaceutical and agricultural applications.

## Figures and Tables

**Table 1:** Antibiotics used for detection of Multidrug-Resistant Bacteria.

S. No	Antibiotics	Family
1	Imipenem	Carbapenem
2	Vancomycin	Glycopeptide
3	Toxobactam	Penicillin
4	Ceftriaxone	Cephalosporin
5	Clarithromycin	Macrolide
6	Ciprofloxacin	Fluoroquinolones
7	Azithromycin	Macrolide
8	Erythromycin	Macrolide
9	Amoxicillin	Penicillin
10	Gentamicin	Aminoglycoside

**Table 2:** Comparative Physicochemical Analysis of Rhizosphere of *Withania coagulans* and *Justicia adhatoda.*

S. No	Physicochemical Characteristics	*Withania coagulans*	*Justicia adhatoda*
1	Clay	12%	10%
2	Slit	26%	26%
3	Sand	54%	64%
4	Organic Matter	1.03%	1.06%
5	Nitrogen	0.052 mg/kg	0.053 mg/kg
6	Phosphorus	25.7 mg/kg	71.5 mg/kg
7	Potassium	108 mg/kg	78 mg/kg

**Table 7:** Bacterial Characterization by Gram Stain and Growth Characteristics.

S. No	Gram Staining	Microscopy			Culture Characteristics on Nutrient Agar
		Color	Shape	Arrangement	
1	-	Pink	Rods	Pairs, Singles	Large, greyish white, smooth, opaque or translucent colonies
2	-	Pink	Rods	Single or pairs	Irregular, greenish blue, smooth, opaque-translucent colonies
3	-	Pink	Rods	Single, pairs or short chains	Circular, Greyish white, mucoid, opaque-translucent colonies
4	-	Pink	Rods	Pairs, singles	Circular, Greyish white, smooth, opaque-translucent colonies
5	-	Pink	Rods	Pairs, singles	Circular, Greyish white, smooth, translucent colonies
6	-	Pink	Rods	Pairs, singles	Smooth, Greyish white, cauliflower like colonies
7	+	Purple	Spherical/cocci	Clusters, single	Round, convex, smooth, golden yellow, opaque colonies
8	+	Purple	Spherical/cocci	Grape-like clusters	Circular, cream-colored, smooth with transparent borders colonies

**Table 8:** Biochemical Profiling of Diabetic Foot Ulcer Bacteria.

S. No	Biochemical Tests									Bacteria
	Enzyme Based			Media Based						
	Oxidase	Catalase	Coagulase	TSI			Citrate	Indole	Urease	
				Slant/Butt	Gas	H_2_S				
1	-	+	-	Acid/Acid	+	-	-	+	-	*E. coli*
2	+	+	-	Alkaline/Alkaline	+	-	+	-	-	*P. aeruginosa*
3	-	+	Nil	Acid/Acid	+	-	+	-	+	*K. pneumoniae*
4	-	+	Nil	Alkaline/Acid	+	-	-	-	-	*Shigella*
5	-	+	Nil	Alkaline/Acid	-	+	-	-	-	*S. typhi*
6	-	+	Nil	Acid/Acid	+	-	+	-	-	*E. aerogenes*
7	-	+	+	Acid/Acid	-	-	+	-	+	*S. aureus*
8	-	+	-	Alkaline/Acid	+	+	-	-	+	*S. epidermidis*

Key: “-“ = Negative, “+” = Positive, Alkaline/Acid (Red slant/Yellow butt) = Dextrose fermentation, Acid/Acid (Yellow slant/Yellow butt) = Dextrose, Lactose, Sucrose fermentation, Alkaline/Alkaline (Red slant/Red butt) = Absence of carbohydrate fermentation, Gas = Production of CO_2_ and H_2._

**Table 9:** Antibiotic Resistance Profile of Bacterial Isolates from Diabetic Foot Ulcers.

Bacteria	Total Antibiotics Tested	No. Resistant	% Resistance
*E. coli*	11	5	45%
*P. aeruginosa*	11	10	91%
*K. pneumoniae*	11	6	55%
*Shigella*	11	7	64%
*S. typhi*	11	5	45%
*E. aerogenes*	11	8	73%
*S. aureus*	11	2	18%
*S. epidermidis*	11	2	18%

## Data Availability

The original contributions presented in this study are included in the article. Further inquiries can be directed to the corresponding authors.

## Abbreviations

The following abbreviations are used in this manuscript:

GC-MS	Gas chromatography-mass spectrometry
*A. luchuensis*	Aspergillus luchuensis
*A. flavus*	Aspergillus flavus
PDA	Potato Dextrose Agar
DNA	Dioxyribonucleic acid
RNA	Ribonucleic acid
MDR	Multi Drug Resistance
